# Research progress on antitumor activity of XRP44X and analogues as microtubule targeting agents

**DOI:** 10.3389/fchem.2023.1096666

**Published:** 2023-03-01

**Authors:** Chao Wang, Lingyu Shi, Shanbo Yang, Jing Chang, Wenjing Liu, Jun Zeng, Jingsen Meng, Renshuai Zhang, Dongming Xing

**Affiliations:** ^1^ Cancer Institute, The Affiliated Hospital of Qingdao University, Qingdao University, Qingdao, China; ^2^ School of Basic Medicine, Qingdao University, Qingdao, China; ^3^ School of Life Sciences, Tsinghua University, Beijing, China

**Keywords:** colchicine binding site inhibitors, XRP44X, structural modification, SAR, antitumor activity

## Abstract

Cancer threatens human health and life. Therefore, it is particularly important to develop safe and effective antitumor drugs. Microtubules, the main component of cytoskeleton, play an important role in maintaining cell morphology, mitosis, and signal transduction, which are one of important targets of antitumor drug research and development. Colchicine binding site inhibitors have dual effects of inhibiting proliferation and destroying blood vessels. In recent years, a series of inhibitors targeting this target have been studied and some progress has been made. XRP44X has a novel structure and overcomes some disadvantages of traditional inhibitors. It is also a multifunctional molecule that regulates not only the function of tubulin but also a variety of biological pathways. Therefore, the structure, synthesis, structure-activity relationship, and biological activity of XRP44X analogues reported in recent years were summarized in this paper, to provide a useful reference for the rational design of efficient colchicine binding site inhibitors.

## 1 Introduction

The International Cancer Research Institute (IARC) released the latest cancer burden data in 2020, which has been used to estimate the latest incidence rate, mortality rate, and cancer development trend of 36 cancer types in 185 countries. Statistics show that 19.29 million people are newly diagnosed with cancer and nearly 10 million people die. Cancer seriously affects human health and threatens human life, and has become one of the major public health problems in the world (Sung et al., 2021; Wang et al., 2021c).

**FIGURE 1:**
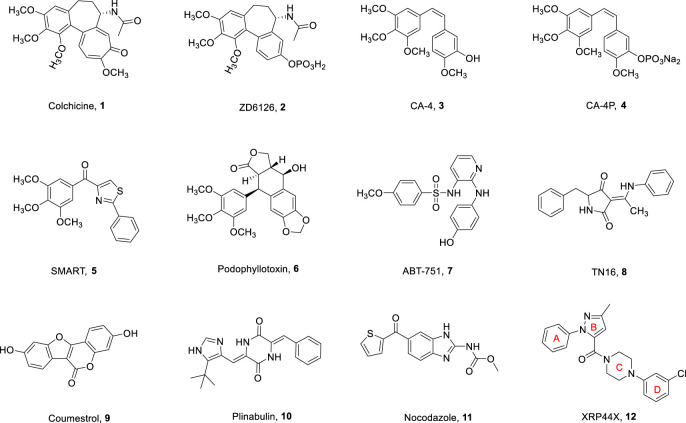
Chemical structures of some colchicine binding site inhibitors.

Microtubules are the main components of eukaryotic cytoskeleton, consisting of *α*-tubulin and *β*-tubulin, which have dynamic characteristics of depolymerization and polymerization, and play a vital role in cell division, morphological maintenance, and intracellular transport (Wieczorek et al., 2016). Interfering with the dynamic balance of microtubules by inhibiting tubulin polymerization or blocking tubulin decomposition will prevent the normal function of microtubules and eventually lead to cell death (Cao et al., 2018). Given their fundamental role in cell function and growth, microtubules have become an attractive target for the development of antitumor drugs (Chaudhary et al., 2016). Microtubule targeting agents (MTAs) can generally be divided into tubulin depolymerization inhibitors and tubulin polymerization inhibitors, which disrupt microtubule dynamics by binding to tubulin and causing cell death. MTAs are currently known to interact with tubulin through several binding sites: the vinca, laulimalide, maytansine, pironetin, taxane, and colchicine sites (Alpizar-Pedraza et al., 2022; Pal et al., 2022). Clinically applied microtubule targeting agents have been very successful in binding to vinca or taxane sites. These drugs are very effective, but some inhibitors targeting the vinca and taxane sites exhibit multidrug resistance, such as P-glycoprotein-mediated resistance. Colchicine binding site inhibitors (CBSIs) exert their biological activities by inhibiting the important process of tubulin assembly (Eissa et al., 2021). Because of their simple structure, wide therapeutic index, and significant ability to overcome clinically related multidrug resistance, they have attracted much attention in antitumor treatment (Cermak et al., 2020; Hagras et al., 2021). In recent decades, many tubulin inhibitors targeting colchicine binding sites (**1–12**, Figure 1) have been developed and modified (Gracheva et al., 2020; Xia et al., 2020). Most of these CBSIs with different backbones have strong antiproliferative activity against a variety of human tumor cell lines.

XRP44X is an aryl pyrazole derivative developed by *wasylyk et al.*. As a new tubulin polymerization inhibitor, XRP44X significantly inhibits the polymerization of tubulin by interacting with the colchicine binding site and shows effective cytotoxic activity on a variety of cancer cell lines at low nanomolar concentrations (Wasylyk et al., 2008). As shown in Figure 1, XRP44X has four rings: A-ring, B-ring, C-ring, D-ring, and a carbonyl bond between B-ring and C-ring. Importantly, XRP44X has a novel structure and overcomes the shortcomings of some traditional CBSIs. Moreover, XRP44X is a multifunctional molecule that not only interacts with tubulin but also has a variety of biological activities. For example, *Cheng et al.* found that XRP44X analogues can be used as anti-influenza drugs targeting viral nucleoprotein (Cheng et al., 2012). Therefore, the structural skeleton of XRP44X has attracted extensive attention. Many pharmaceutical chemists have developed some compounds with potential antitumor activity by modifying the structure of XRP44X for further research. In recent years, some interesting XRP44X analogues have been reported by modifying the B-ring (five-membered and six-membered heterocyclic hybrids), C-ring (aryl piperazinyl modified), and the substituted phenyl of A- and D-rings. Therefore, we have comprehensively and systematically reviewed the design, synthesis, structure-activity relationship (SAR), and pharmacological activities of XRP44X analogues modified by different heterocycles reported in recent years, to provide research ideas for obtaining CBSIs with better antitumor activity and higher stability.

## 2 Biological effects of XRP44X

### 2.1 Interaction with tubulin

**FIGURE 2:**
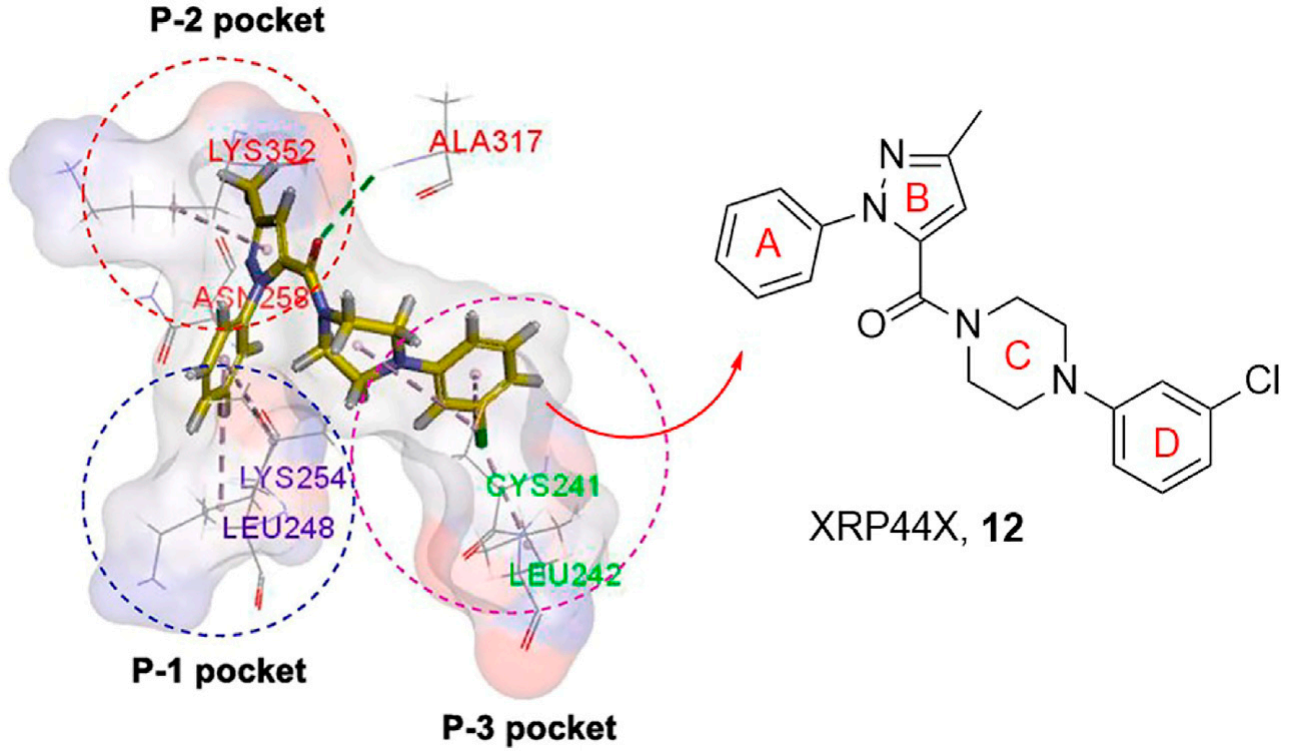
The binding mode of XRP44X against tubulin.

XRP44X is an outstanding tubulin polymerization inhibitor. The binding mode of XRP44X or its analogues with tubulin is shown (Figure 2), the binding domain can be divided into P-1, P-2, and P-3 pockets (Wang et al., 2021b). First, the hydrogen bond is formed through the carbonyl group with the amide nitrogen of Ala*β*317 and acts as an anchor to place XRP44X in its proper position in the binding pocket. A-ring can match in P-1 pocket Lys*β*254 and Leu*β*248 binding and enhance the van der Waals interaction between XRP44X and tubulin. Asn*β*258 and Lys*β*352 in P-2 pocket amino acid residues can form hydrogen bond interaction with the B-ring, and also enhance its van der Waals force. The C-ring and D-ring extend and interact with the hydrophobic P-3 pocket consisting of Leu*β*242, Leu*β*252, and Cys*β*241. The above studies clearly indicate docking patterns between XRP44X and tubulin.

Further studies showed that tubulin polymerization inhibitors can destroy the tubulin skeleton and induce cell cycle arrest by regulating cyclin B1, cyclin-dependent kinase CDK1, etc., (Liang et al., 2021). And they induce apoptosis by regulating the levels of apoptosis-related proteins such as Bax, P53, PARP, and cleaved-Caspase 9. Besides, tubulin polymerization inhibitors reduce mitochondrial membrane potential and increase the level of reactive oxygen species (ROS) in cells (Yan et al., 2022). Excessive ROS can induce oxidative modification of lipids, proteins, or DNA, leading to oxidative stress-induced apoptosis (Circu and Aw, 2010). Tubulin polymerization inhibitors play an antitumor role through the above pathways.

### 2.2 Interaction with other biological targets

In addition to interacting with tubulin, XRP44X also regulates multiple biological pathways. Transcription factors are powerful drivers of cell transformation and play a significant role in tumorigenesis (Feng et al., 2014). Studies have found that Elk3 of the ETS family is a proto-oncogene transcription factor and a potential therapeutic target for many cancers, including breast cancer and liver cancer. Elk-3 can be phosphorylated and activated by the Ras-Erk signal pathway, and participate in angiogenesis, wound healing, tumor growth, cell adhesion, migration, and invasion (Heo et al., 2015; Kong et al., 2016; Lee et al., 2017). *Wasylyk et al.* discovered XRP44X, an active inhibitor of Elk3 transcription factor, in small molecule library screening. XRP44X was found to inhibit the phosphorylation of Elk3 induced by fibroblast growth factor 2 (FGF-2) through Ras-Erk signaling. It has also been found to bind to colchicine binding sites of tubulin, depolymerize microtubules, change actin skeleton, stimulate cell membrane foaming, affect transcriptional regulation, block cell cycle processes, and microvascular germination (Wasylyk et al., 2008). *Semenchenko et al.* conducted a preliminary evaluation of the effect of XRP44X on the tumor in three preclinical mouse models and found that XRP44X inhibits tumor growth and metastasis through mechanisms involving Elk3. These results encourage further research on XRP44X, which could be useful in the development of antitumor drugs.

Natural Killer (NK) cells are cytotoxic lymphocytes that can kill virus-infected cells and cancer cells. *Kim* and *Park* found that pyrazole derivative XRP44X stimulated NK cells to enhance cytotoxicity to breast cancer cells. In the presence of XRP44X, natural killer cells showed no obvious apoptosis or impaired cell cycle progression. But XRP44X stimulates interferon γ and activates the c-JUN N-terminal kinase (JNK) signal pathway to enhance the cytotoxicity mediated by NK cells (Kim and Park, 2020). *Chen et al.* also found that XRP44X and CA-4 induced the depolymerization of tubulin and G2/M phase arrest of the cell cycle through early activation of the JNK pathway (Chen et al., 2012). This study revealed that XRP44X is not only identified as a remarkable CBSI but also acts as an immune stimulator to enhance the cytotoxicity of NK cells to kill cancer cells. This provides a new strategy for the combination of chemotherapy and immunotherapy in the treatment of cancer.

**FIGURE 3:**
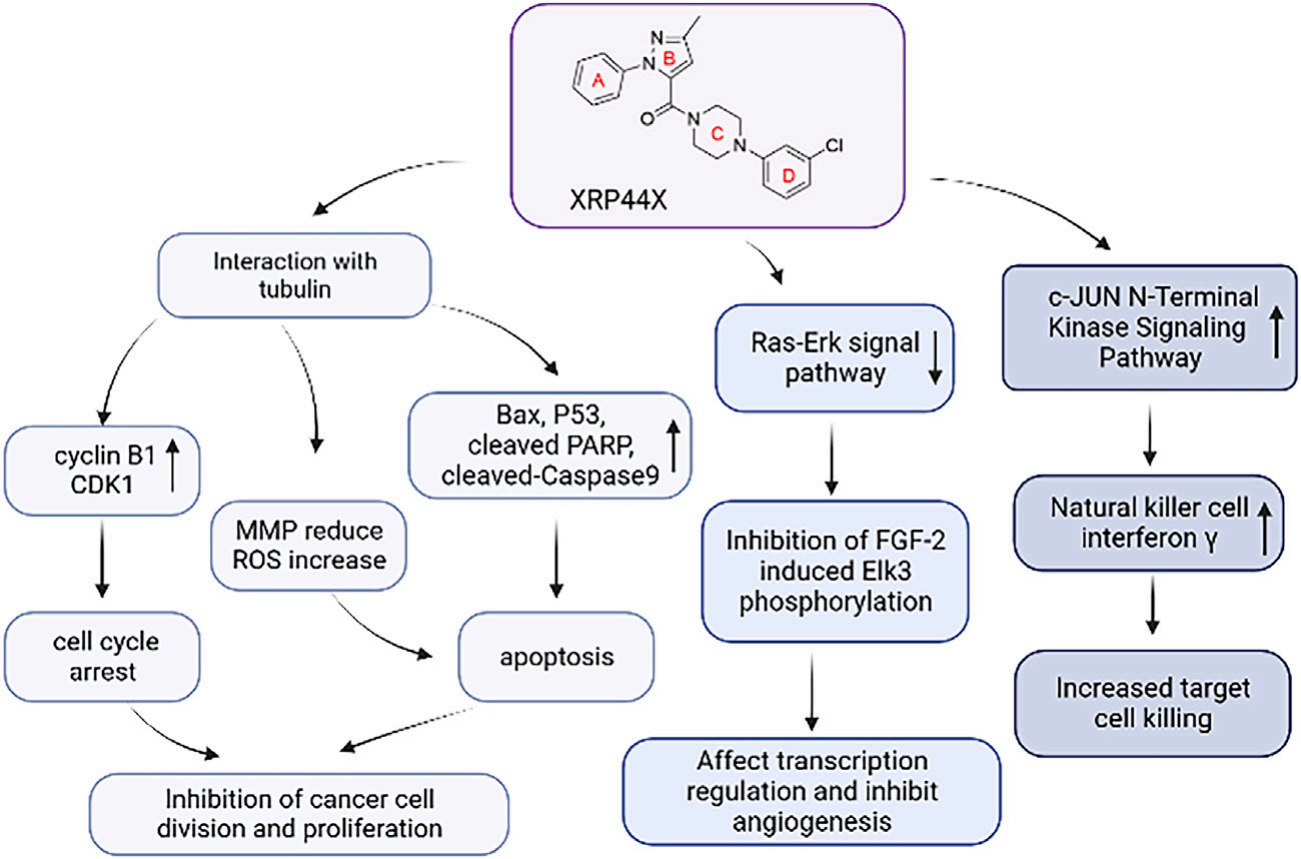
The mechanism of action of XRP44X.

As shown in Figure 3, the above studies confirm that XRP44X can exert biological effects through multiple pathways. Although XRP44X has proven to be a promising lead compound for tubulin polymerization inhibitors, we still need to pay attention to the multi-target compounds to develop drugs that can be used clinically.

## 3 B-ring modified XRP44X analogues

### 3.1 Five-membered heterocyclic hybrids

#### 3.1.1 Thiadiazole

**FIGURE 4:**
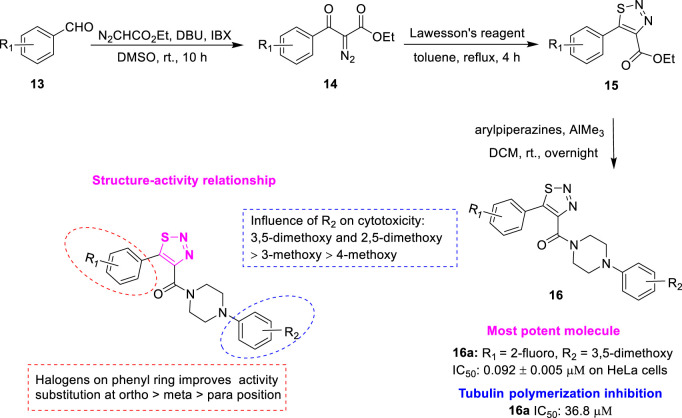
Synthetic strategy for compound **16** and SAR.

*Wang et al.* designed a series of 5-aryl-4-(4-aryl piperazine-1-carbonyl)-1,2,3-thiadiazole **16** (Figure 4) as new tubulin polymerization inhibitors by replacing the pyrazole portion of XRP44X with the 1,2,3-thiadiazole fragment containing the hydrogen-bond acceptors (Wang et al., 2021b). Initially, the substituted aromatic aldehydes **13** were reacted with ethyl diazoacetate to give the corresponding ethyl 2-diazo-3-oxo-3-arylpropanoates **14**. Subsequently, the reaction was commenced using **14** and Lawesson’s reagent to yield the key intermediate ethyl 5-aryl-1,2,3-thiadiazole-4-carboxylates **15**. Finally, treatment with the corresponding arylpiperazine in the presence of trimethylaluminum afforded the target compounds **16**. The *in vitro* antiproliferative activity against human cancer cell lines (SGC-7901, A549, HeLa) of the synthesized compounds was further evaluated. Among the target compounds, compound **16a** (R_1_ = 2-fluoro, R_2_ = 3,5-dimethoxy) showed the best antiproliferative activity against HeLa cells (IC_50_ = 0.092 ± 0.005 μM), similar to the positive control colchicine (IC_50_ = 0.086 ± 0.008 μM on HeLa cells). Compound **16a** can effectively inhibit tubulin polymerization (IC_50_ = 36.8 μM). SAR showed significant changes in the activity of all halogenated compounds compared to the non-halogenated compounds, suggesting that electronegativity is crucial for the A-ring. Moreover, halogens on the A-ring exhibited an order of potency being ortho- > meta- > para-substituted generally. It is noteworthy that compounds with substituents at the para-substituted of the A-ring almost lost antiproliferative activity. In addition, compounds with OCH_3_ on the D-ring interposition substitution showed higher activity than those with the para-substitution, with di-meta-substituted compounds showing increased activity. These results suggest that the volume and lipophilicity of the substituents on the D-ring are important factors affecting the activity. Molecular docking results showed that compound **16a** may similarly interact with tubulin to XRP44X. In addition, 1S and 3N of 1,2,3-thiadiazole of compound **16a** may form additional hydrogen bonding interactions. This new binding mode in the P-2 pocket was first explored in the design of XRP44X analogues.

#### 3.1.2 Triazole

**FIGURE 5:**
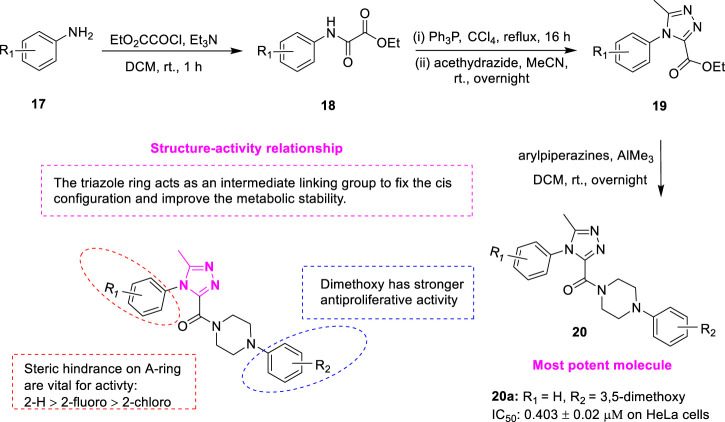
Synthetic strategy for compound **20** and SAR.

1,2,4-triazoles is a five-membered aromatic heterocycle containing three nitrogen atoms, which has been of wide interest due to their ease of preparation and the presence of numerous potential pharmacological properties such as antituberculosis (Kahveci et al., 2014), antiviral activity (Gujjar et al., 2009), antioxidant (Flefel et al., 2013) and antitumor activity (Romagnoli et al., 2014). *Wang et al.* designed and synthesized a series of novel XRP44X analogues **20** (Figure 5) containing 1,2,4-triazoles and investigated antitumor activity and mechanism of action (Wang et al., 2020). The ethyl 2-oxo-2-(arylamino) acetates **18** were synthesized by the commercially available aromatic amines **17** as the starting materials and ethyl oxalate. Compound **18** was reacted with triphenylphosphine to afford the corresponding (E)-ethyl-2-chloro-2-(arylimino) acetates, which further added acethydrazide to yield the key intermediate 5-methyl-4-aryl-4H-1,2,4-triazole-3-carboxylates **19**. In the presence of trimethylaluminium, **19** were allowed to react with the corresponding aryl piperazines, affording the final compounds **20**. The biological assessment suggested that compound **20a** exhibited the strongest antiproliferative activity (IC_50_ = 0.403 ± 0.02 μM) on Hela human cancer cells. The IC_50_ of positive control CA-4 on HeLa cells was 0.063 ± 0.015 μM. The stronger activity was attributed to the introduction of the triazole ring as an intermediate linker group, thus fixing the cis-configuration with anti-proliferative activity and improving metabolic stability. The steric hindrance played a key role in the A-ring. For example, compared with 2-chloro, the activities of 2-H and 2-fluoro were greatly improved. The activity of the binary substituted methoxy compounds on the D ring is stronger. Compound **20a** effectively inhibited tubulin polymerization, interfered with the normal morphology of intracellular microtubules, significantly inhibited the migration of Hela cells, and caused cell cycle arrest in the G2/M phase in a dose- and time-dependent manner. To investigate the potential binding modes of the target compounds, molecular docking studies were performed using Discovery Studio 3.0 software. It was found that the binding orientations of XRP44X and compound **20a** superimposed well with each other. There are hydrogen bonds between Ala*β*317 and the carbonyl group of XRP44X and compound **20a**, respectively. In addition, the 1N of the 1,2,4-triazole of compound **20a** established a critical hydrogen bond with the amino acid residue Lys*β*352. The docking results indicated that compound **20a** may show its biological activity through the combination with the colchicine site.

**FIGURE 6:**
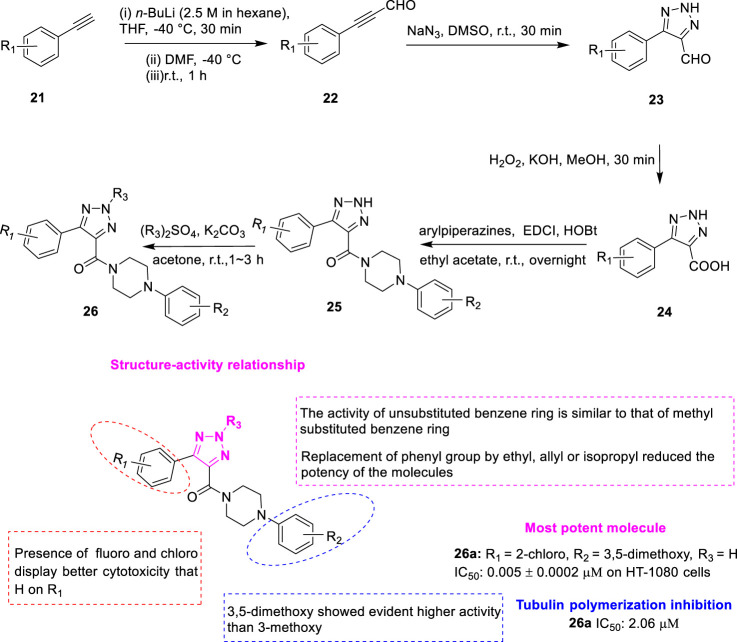
Synthetic strategy for compounds **25**, **26,** and SAR.

*Wu et al.* discovered two series of aryl triazole analogues as a result of structural modifications of the lead compound XRP44X. Namely, the first class of compounds **25** (Figure 6) with 1,2,3-triazole nitrogen atom unsubstituted and the second class of compounds **26** with 1,2,3-triazole N-2 position hydroalkylated (Wu et al., 2017). Arylacetylene analogues **21** reacted with *N,N*-dimethylformamide to give arylpropionaldehyde analogues **22**. Then azide-alkyne 1,3-dipolar cycloadditions were added to give 5-aryl-2*H*-1,2,3-triazole-4-carbaldehyde **23**, which was then oxidized with hydrogen peroxide to 5-aryl-2*H*-1,2,3-triazole-4-carboxylic acid **24**. Finally, the corresponding arylpiperazine analogues were added to give the desired compound **25** by amidation reaction. The second type of compound **26** could be obtained by the selective hydrocarbylation of the first type of compound **25** at the N-2 position of 1,2,3-triazole. The antiproliferative activity of the synthesized compounds against cancer cells was investigated using an MTT assay. The results showed that most of the compounds exhibited strong antiproliferative activity at submicromolar or nanomolar concentrations, which justified the introduction of the 1,2,3-triazole fragment. Among the designed compounds, **26a** (R_1_ = 2-chloro, R_2_ = 2-dimethoxy, R_3_ = H) showed an IC_50_ value of 5–52 nM towards all 3 cell lines (A549, HT-1080, and SGC-7901), which was comparable to CA-4 (7–48 nM). Compound **26a** (IC_50_ = 2.06 μM) highly inhibited tubulin aggregation *in vitro*, stronger than the positive control CA-4. Colchicine competitive binding assays further demonstrated that compound **26a** is a colchicine binding site inhibitor. SAR showed no significant change in antiproliferative activity for all methylated products compared to the corresponding non-hydrocarbonylated compounds, such as compound **26a**. However, the activity was significantly reduced as the bulky alkyl group was introduced. These phenomena suggest that hydrocarbonylation at the N-2 position is not required. Secondly, the effect of the substituents of the benzene ring attached to the piperazine ring was examined, and the results indicated that the spatial effect of the substituents was more important than the electronegativity effect. In addition, the di-meta-substituted compounds have better antiproliferative activity. Finally, the effect of substituents on the aryl ring directly linked to the 1,2,3-triazole core was investigated. Most substitutions failed to improve activity, but electronegativity and position were found to be extremely important for activity. Molecular docking models showed that both **25** and **26** overlapped well with XRP44X. The carbonyl group provides a hydrogen bond Ala*β*317 to these three ligands. Although **25** lacks a methyl group, the 2H on its 1,2,3-triazine ring can provide a hydrogen bond to Thr*β*353.

#### 3.1.3 Tetrazole

**FIGURE 7:**
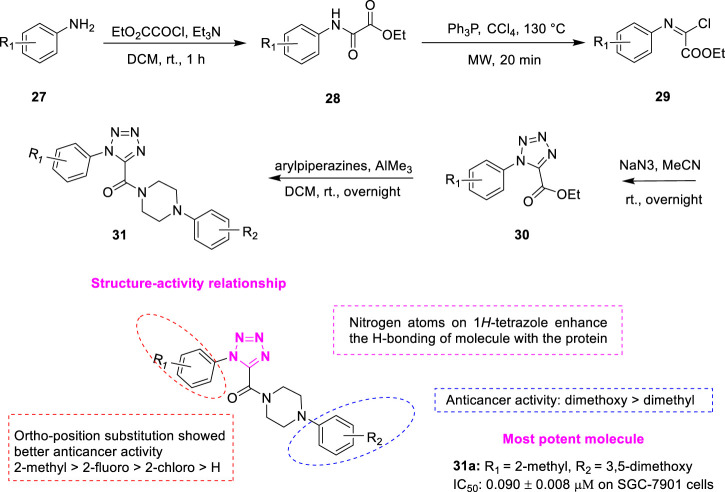
Synthetic strategy for compound **31** and SAR.

In the last decade, compounds containing 1*H*-tetrazole scaffolds have attracted great interest due to unique chemical structures and broad-spectrum biological properties including antitumor activity. For example, 1-(3,4,5-trimethoxyphenyl)-5 (4-ethoxyphenyl)-1*H*-tetrazole was designed as a tubulin inhibitor that showed low IC_50_ values at the nanomolar level (Romagnoli et al., 2012). *Wang et al.* designed a series of novel XRP44X analogues **31** (Figure 7) by introducing the hydrogen-bonded receptor 1*H*-tetrazole as the B-ring of XRP44X (Wang et al., 2021a). Condensation of the substituted aromatic amines **27** and ethyl oxalate gave corresponding intermediates **28**, which reacted with triphenylphosphine to afford (*E*)-ethyl-2-chloro-2-(arylimino) acetates **29**. Compound **29** reacted with sodium azide to get the key intermediates ethyl 1-aryl-1*H*-tetrazole-5 carboxylates **30**. Further reaction of **30** with different arylpiperazines in the presence of trimethylaluminium produced the target compound **31**. These compounds were found to show good growth inhibitory activity against a series of human cancer cells (SGC-7901, A549, and HeLa). Among them, compound **31a** was the most potent compound among all target compounds against the three cancer cell lines (IC_50_ = 0.090 ± 0.008 μM on SGC-7901 cells). The IC_50_ of positive control CA-4 was 0.034–0.070 μM. A closer look revealed that the introduction of substituents in the neighboring positions of the R_1_ significantly enhanced the antiproliferative effect. The overall preference order of the ortho-position is as follows: 2-methyl > 2-fluoro > 2-chloro > H. In addition, the compounds of R_2_ showed significant antitumor activity in the case of 3,5-dimethoxy phenyl dense. Molecular docking studies revealed that there is a hydrogen bond between Ala*β*317 and the carbonyl group of **31a**, which has a similar conformation to XRP44X in the binding pocket, and two other hydrogen bonds were observed between Asn*β*258, Lys*β*352, and 1*H*-tetrazole. In addition, the study of drug-like properties showed that compound **31a** has a lower lipid-water partition coefficient, and may have better water solubility than XRP44X. **31a** has six hydrogen bond receptors, far more than XRP44X, which helps to reduce the interaction between the compound and the site.

#### 3.1.4 Oxazole, thiazole, and isoxazole

**FIGURE 8:**
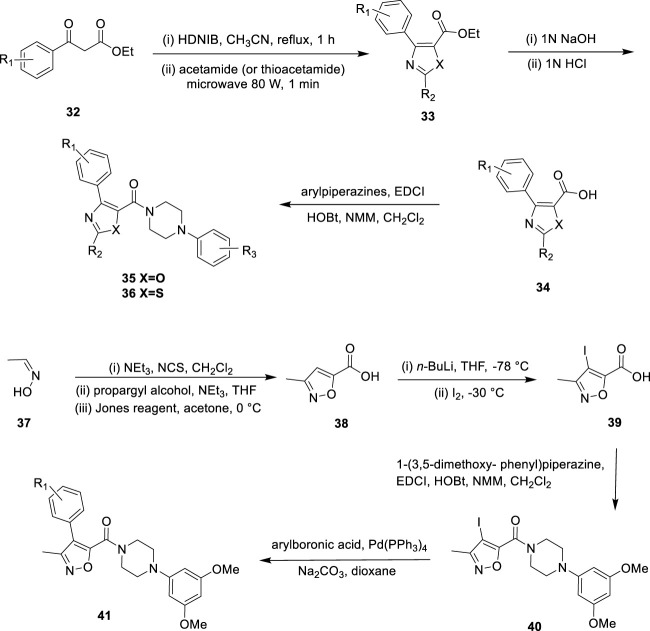
Synthetic strategy for compound **35**, **36**, and **41**.

**FIGURE 9:**
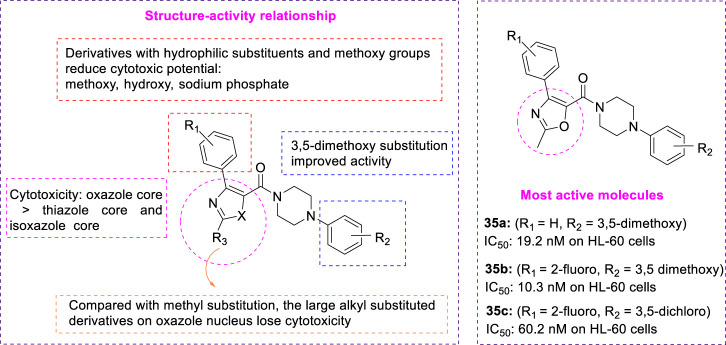
SAR and active molecules of aryloxazole analogues.

To develop a new class of antitumor drugs with dual effects: it can not only destroy the tumor vascular system but also prevent mitosis. *Choi et al.* reported the design, synthesis, and biological evaluation of aryl oxazole, thiazole, and isoxazole analogues as dual-acting antitumor agents, and proposed a new core complete SAR study to determine the basic structural properties required for dual action (Choi et al., 2013). Ethyl 3-oxy-3-phenylpropanoate reacted with [hydroxy (2,4-dinitrobenzene sulfonyl oxy) iodol] benzene and acetamide to form heterocyclic core **33** (Figure 8). Then, **33** was hydrolyzed to obtain fragment **34**, and various aryl piperazines were added for a polypeptide coupling reaction to obtain the desired aryl oxazole **35** and thiazole analogues **36**. After treatment of (*E*)-acetaldehyde oxime with triethylamine and *N*-chlorosuccinimide, propargyl alcohol was added to obtain isoxazole cores. The primary alcohol group was then converted to carboxylic acid to give the isoxazole fragment **38**. Iodination of intermediate **38** with *n*-BuLi and iodine to form compound **39**, which reacted with 1-(3,5-dimethoxy phenyl) piperazine to afford intermediate **40**. The reaction was performed by aryl boronic acids to yield a series of methyl-substituted isoxazole analogues **41**. *In vitro*, HL-60 (leukemic cells) and HUVEC (human umbilical vein endothelial cells) were used to test the antiproliferation activity and tumor vessel destruction activity of all synthetic compounds. Several compounds with aryl piperazinyl oxazole nuclei showed good cytotoxicity. **35a** (R_1_ = H, R_2_ = 3,5-dimethoxy, IC_50_ = 19.2 nM on HL-60 cells) and **35b** (R_1_ = 2-fluoro, R_2_ = 3,5-dimethoxy, IC_50_ = 10.3 nM on HL-60 cells) (Figure 9) showed outstanding antitumor activity in comparison with CYT997 (IC50 = 112.3 nM) as reference. SAR showed that the potency of all analogues containing thiazole or isoxazole nuclei was significantly reduced, and it was determined that the antitumor activity of compounds containing oxazole nuclei was crucial. *Choi et al.* studied the effect of substituents on the benzene ring directly connected to the oxazole nucleus and found that analogues with hydrophilic substituents (such as hydroxide, and sodium phosphate) and methoxy were less effective than their unsubstituted counterparts. This indicated that van der Waals interaction may play an important role in this specific binding region. Compared with the parent compound without any substituent, only the analogues with 3,5-dimethoxy at the R_2_ position have slightly improved activity, which indicates that there may be hydrogen bond interaction in this region. *Choi et al.* replaced the methyl groups on the oxazole nucleus with different alkyl groups and found that as the size of the substituents became larger, these analogues completely lost their cytotoxicity. These suggested that the oxazole-binding region can only accommodate a small substitution basis for good interaction.

Considering the biological activity and metabolic stability *in vitro*, a simple tumor xenotransplantation model (HCT-116, human colorectal cancer) was used to evaluate the antitumor activity of the two most effective compounds **35b** (R_1_ = 2-fluoro, R_2_ = 3,5-dimethoxy) and **35c** (R_1_ = 2-fluoro, R_2_ = 3,5-dichloro) *in vivo*. **35b** has better cytotoxicity and vascular destruction activity than **35c**. However, compound **35c** showed significant microsome stability (82.3% for humans and 49% for mice), while compound **35b** seemed only moderately stable in liver microsomes (15% for humans and 16.6% for mice). These two compounds inhibited tubulin polymerization at low concentrations. Interestingly, although **35c** is much less potent *in vitro* than **35b**, it has a greater impact on tumor growth *in vivo*. Compound **35c** effectively reduced tumor growth (42.3% in size) at a dose of 100 mg/kg. This compound will be an excellent clue to the discovery of effective double-acting agents.

### 3.2 Six-membered heterocyclic hybrids

**FIGURE 10:**
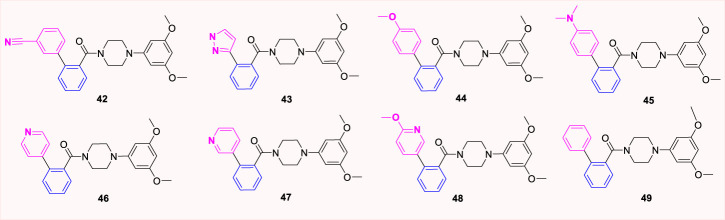
Structure of six-membered heterocyclic hybrid analogues.

*Betzemeier et al.* designed and developed a series of aryl piperazine benzamide analogues used as antitumor drugs (Betzemeier et al., 2006). Among them, we found many XRP44X analogues with six-membered benzene ring as B-ring. A series of (1,1′-biphenyl)-2-yl [4-(3,5-dimethoxyphenyl)piperazin-1-yl] methanone analogues (**42–49**, Figure 10) were prepared according to the method described in the patent, and cytotoxicity and tubulin polymerization inhibition tests were performed *in vitro*. The results show that the IC_50_ values of the reported XRP44X analogues are lower than 10 μM and are confirmed as tubulin polymerization inhibitors. Interestingly, XRP44X analogues inhibit angiogenesis and affect the proliferation of abnormal cells, which can be used in the treatment of retinal vascularization and arthritis.

## 4 Other substituted analogues

**FIGURE 11:**
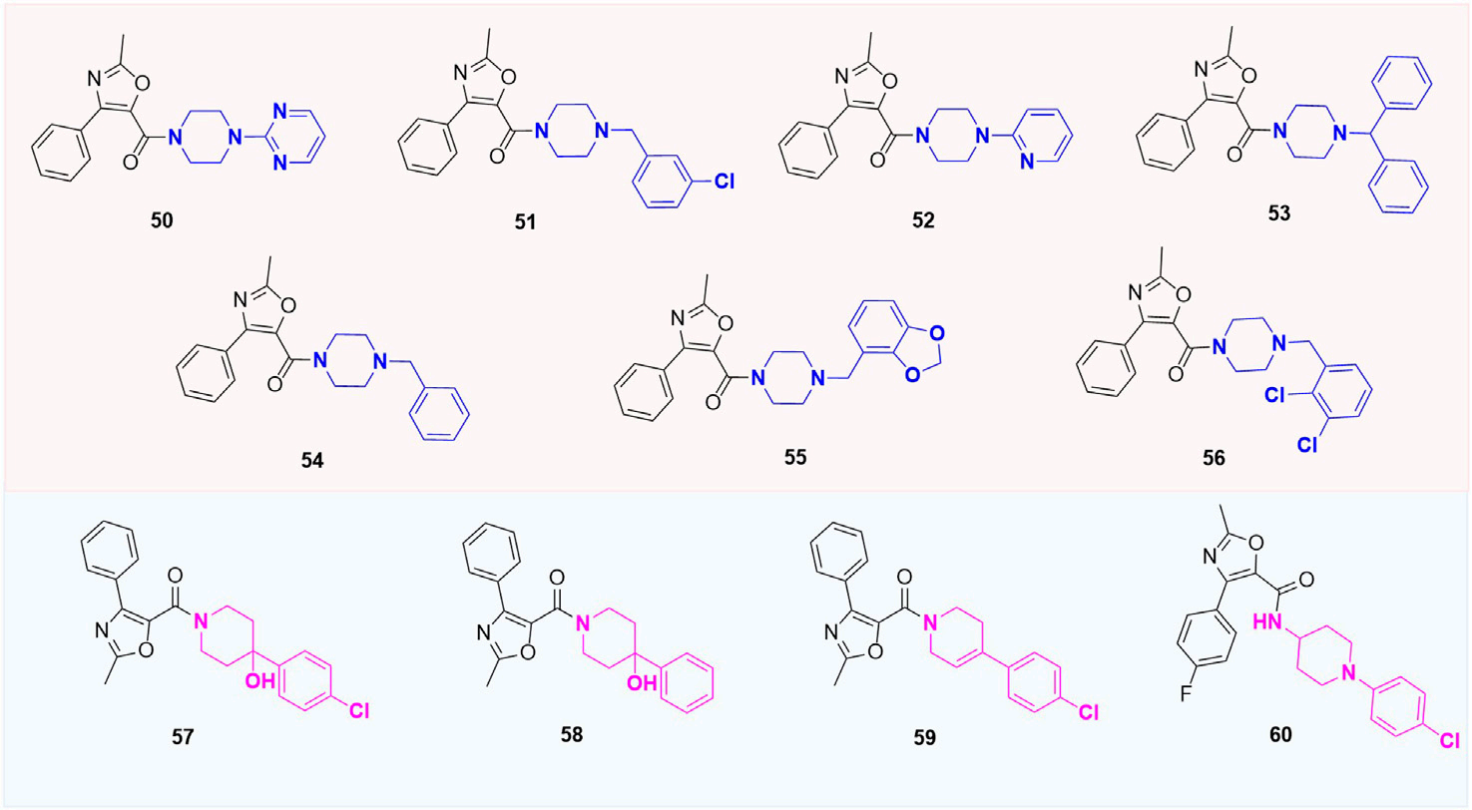
Structure of aryl piperidine analogues.

**FIGURE 12:**
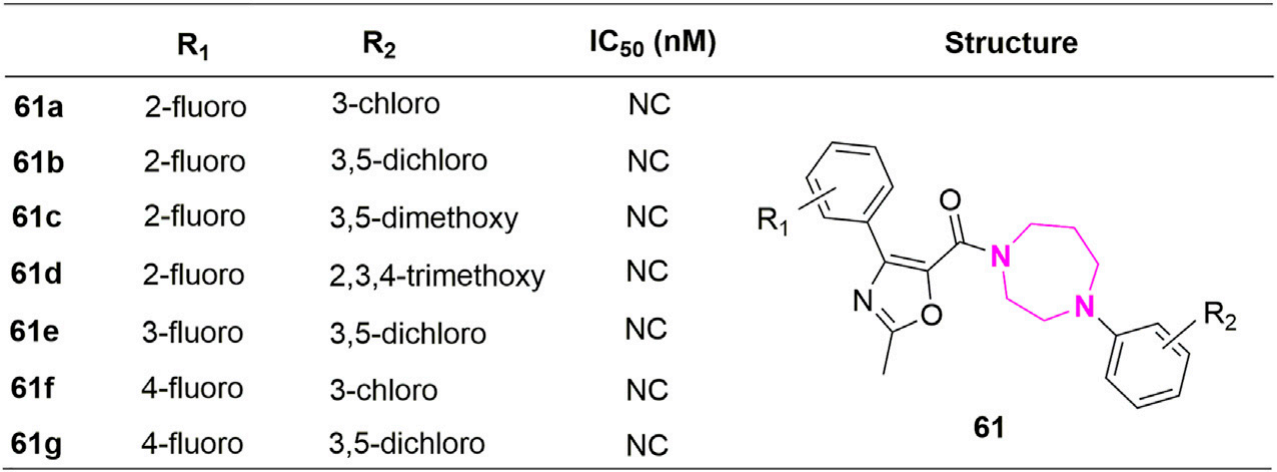
Structure and activity of homopiperazine analogues.

Except for the modification of the B-ring, some XRP44X analogues modified with other rings were also reported. *Pae et al.* substituted aryl piperazine groups on oxazole with various heterocycles (aryl piperidine and homopiperazines) and developed several series of (2-methyl-4-phenyloxazole-5-yl) ketone analogues (**50–60**, Figure 11) and (2-methyl-4-phenyloxazol-5-yl) (phenylhomo-piperazin-1-yl) methanone analogues (**61a-61g**, Figure 12). The cytotoxicity of all target compounds on human leukemia cells (HL-60) was tested. However, all the modified compounds in both series completely lost cytotoxicity, further illustrating the importance of the two nitrogen atoms in piperazine-formed hydrogen bond interactions in this region. Moreover, the spatial arrangement that replaces the aryl seems to be the key to a correct combination.

**FIGURE 13:**
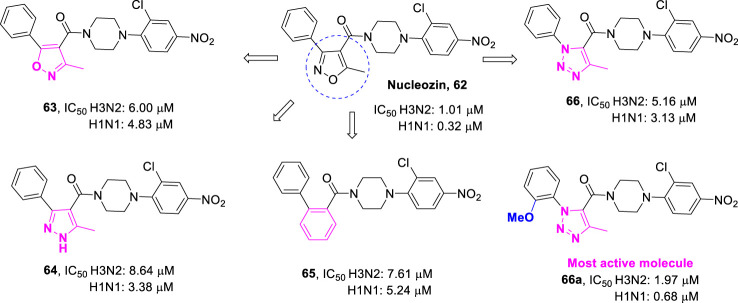
New anti-influenza agents targeting virus nucleoprotein.

Not all XRP44X analogues have been synthesized as antitumor agents. Some have been developed to fight influenza viruses. Influenza virus nucleoprotein (NP) is a new target for the development of anti-influenza drugs. *Kao* and *Su* reported that nucleozin (**62**, Figure 13) is the first small molecule influenza virus nucleoprotein inhibitor (Kao et al., 2010; Su et al., 2010). The results showed that nucleozin could effectively block the nuclear accumulation of influenza nucleoprotein and showed strong anti-influenza virus activity. With compound **62** as the lead compound, *Cheng et al.* preliminarily designed isoxazole-4-formamide (**63**), 1*H*-pyrazole-4-formamide (**64**), 2-benzamide (**65**), and 1*H*-1,2,3-triazole-4-formamide (**66**) as new anti-influenza drugs by using the scaffold jumping strategy (Cheng et al., 2012). The anti-influenza A virus activity was determined by the cytopathic effect protection experiment on Madin-Darby canine kidney (MDCK) cells. By further structural optimization, the anti-influenza activity of these compounds was improved, even comparable to that of compound **62**. One of the most effective compounds **66a** inhibited the replication of various H1N1 and H3N2 influenza A virus strains, with IC_50_ values ranging from 0.7 to 2.0 μM. Compound **66a** also strongly inhibited the replication of H5N1 (RG14), anti-oseltamivir A/WSN/1933 (H1N1, 274Y), and anti-Amartidine A/WSN/33 (H1N1) virus strains, with IC_50_ values in sub micromolar range. Further computational and mechanism studies indicated that **66a** may directly target influenza virus A nuclear protein to inhibit its nuclear accumulation. This research provides a series of new lead compounds for the development of anti-influenza drugs targeting influenza virus nucleoprotein.

Whether used as antitumor drugs targeting microtubules or as anti-influenza drugs, the potential XRP44X analogues are on the way to the clinic. Therefore, it is very necessary to develop new tubulin inhibitors which can be used in clinical treatment.

## 5 Conclusion

In recent years, colchicine binding site inhibitors have attracted much attention due to their targeting specificity and strong antitumor activity. XRP44X is a novel CBSI with novel structure, strong activity, and wide biological role, which is regarded by many researchers as the basis for designing and developing new chemical structures. We comprehensively and systematically reviewed the design, synthesis, structure-activity relationship, pharmacological activity, and various biological effects of the new XRP44X analogues. The research found that the most popular design strategy of XRP44X analogues is to replace the B-ring with other five-membered nitrogen heterocycles (thiadiazole, triazole, tetrazole, thiazole, oxazole, and isoxazole). In addition to five-membered heterocyclic modifications, a series of XRP44X analogues replaced by six-membered heterocyclic rings have been reported on the B-ring. The carbonyl bond between the B- and C-rings was considered to be the basis of tubulin activity. Interestingly, the antitumor activity of the piperazine group on the oxazole ring was greatly reduced after it was replaced by aryl piperidine or homopiperazine groups, indicating the importance of the two nitrogen atoms in piperazine-formed hydrogen bond interaction in this region. From the above studies, it can be seen that the electronegativity of substituted aryl groups on the A ring is essential for antitumor activity. The volume and lipophilicity of the substituted aryl group on the D-ring are important factors affecting the activity. The substituted aryl group of 3, 5-dimethoxy-phenyl group on the D-ring shows significant antitumor activity. Molecular docking studies have shown that XRP44X analogues are well bound to tubulin. XRP44X and its analogues have been prepared as anti-influenza drugs targeting viral nucleoprotein in addition to their antitumor activities. Hence, the design and biological activity of XRP44X analogues still need further study, especially in the aspects of normal cell selectivity, study and simulation of ADMET, evaluation of various tumor models *in vivo*, pharmacokinetics, toxicology, and drug action mechanism. Considering the latest progress described in this review article, it is obvious that the development of XRP44X analogues enriches the library of compounds targeting tubulin, contributes to further structural adjustment, and provides candidate compounds for the development of antitumor drugs.
